# Morin and Morin Semicarbazone Combined with Fucoxanthin Have Potential Anti-Inflammaging Effects Through Modulation of Nrf2/HO-1 System in UVB-Exposed HaCaT Keratinocytes

**DOI:** 10.3390/antiox15050599

**Published:** 2026-05-09

**Authors:** Sara García-Gil, Javier Ávila-Román, Azahara Rodríguez-Luna, Gabriela Rodríguez-García, Rosa E. del Río, Virginia Motilva, Mario A. Gómez-Hurtado, Elena Talero

**Affiliations:** 1 Department of Pharmacology, Faculty of Pharmacy, Universidad de Sevilla, 41012 Seville, Spain; sgarcia18@us.es (S.G.-G.); amrluna@uloyola.es (A.R.-L.); motilva@us.es (V.M.); 2 Faculty of Health Sciences, Universidad Loyola Andalucía, 41704 Seville, Spain; 3 Instituto de Investigaciones Químico Biológicas, Universidad Michoacana de San Nicolás de Hidalgo, Ciudad Universitaria, Morelia 58030, Michoacán, Mexico; gabriela.rodriguez@umich.mx (G.R.-G.); norma.del.rio@umich.mx (R.E.d.R.); mario.gomez@umich.mx (M.A.G.-H.)

**Keywords:** morin, morin semicarbazone, fucoxanthin, photoprotection, UV protection, inflammaging, antioxidant

## Abstract

Ultraviolet (UV) radiation is a main environmental factor responsible for skin damage, leading to oxidative stress, inflammation, and impairment of the skin barrier function. Furthermore, many components in sunscreen may accumulate in aquatic systems, causing environmental pollution. Therefore, the identification of novel natural bioactives that counteract these effects and can be useful as effective adjuvants in sunscreen formulations is of particular interest. Morin (**1**), a natural flavonoid, represents an attractive scaffold for modifications to enhance its biological activity. Herein, we aimed to investigate the effects of combining the flavonoid **1** and its derivative, morin semicarbazone (**2**), with the carotenoid fucoxanthin (FX) on UVB-exposed HaCaT keratinocytes. All compounds exhibited higher radical scavenging activity compared to Trolox. In this cell model, the phenolic–carotenoid combinations provided greater photoprotection than individual compounds, significantly enhancing cell viability and reducing necrosis, FX-**2** emerged as the most potent combination, as evidenced by a marked reduction in reactive oxygen species (ROS) and malondialdehyde (MDA) levels, likely mediated through the activation of the nuclear factor erythroid 2-related factor 2/Heme oxygenase-1 (Nrf2/HO-1) signaling pathway. Furthermore, the tested treatments exerted enhanced anti-inflammatory effects by significantly reducing interleukin-6 (IL-6), cyclooxygenase 2 (COX-2), and matrix metalloproteinase-9 (MMP-9) mediators, with FX-**2** being the most active combination. In conclusion, our findings highlight the protective effects of the combinations of these phenolics with the carotenoid FX against UVB radiation and support their potential application as natural active ingredients in sunscreen formulations.

## 1. Introduction

The integumentary system is the largest and most extensive organ and is considered the first protective barrier against external agents such as pollution, pathogens, or solar radiation. All of these factors can trigger an inflammatory response, leading to different skin pathologies, altering the homeostasis and skin structure, and ultimately generating skin cancer [1,2]. Ultraviolet (UV) radiation is part of the energy coming from the Sun and comprises UVC, UVB, and UVA. In this regard, UVB radiation can reach the epidermis and the superficial dermis barrier, causing oxidative stress, apoptosis, and carcinogenesis [3]. Therefore, skin photoprotection is a crucial healthy habit for the proper maintenance and function of the skin.

The composition of sunscreens is currently being reconsidered because certain ingredients may cause skin discomfort or negative effects on the environment [4]. In this regard, the only approved inorganic UV filters, titanium oxide (TiO_2_) and zinc oxide (ZnO), which act by absorbing, reflecting and scattering UV radiation [5,6,7], have shown a toxic effect on the aquatic environment. This is due to the fact that their nanoparticles may produce reactive oxygen species (ROS) under certain conditions, resulting in toxicity for marine phytoplankton [8]. On the other hand, the main organic UV filters octinoxate, octocrylene, and to a greater extent oxybenzone, whose function is to absorb UV radiation, are generally considered safe for topical use. Nevertheless, adverse events may occasionally occur such as cutaneous toxicity, causing allergic contact and photoallergic reactions [9,10]. Among others, these are the main reasons why some sunscreen ingredients such as organics filters or stabilizing compounds are being gradually phased out of the market by law. In this regard, some countries such as Hawaii (United State of America) and Thailand have had legal restrictions since 2021 on the use of sunscreens containing these ingredients [11]. These measures are largely due to mass tourism in coastal areas near Australia’s Great Barrier Reef (Coral Triangle), where these ingredients accumulate, causing negative effects in marine organisms [12,13]. In these areas, the use of sunscreens free of octinoxate, octocrylene, and oxybenzone is mandatory. For all of these reasons, developing formulations that incorporate natural bioactive ingredients is a main objective for the sunscreen industry, as these products are widely accepted by the population for being more environmentally friendly and skin-safe. Consequently, sunscreens containing natural and biodegradable products capable of enhancing the photoprotective properties are currently under industrial development.

Nowadays, phenolic compounds and carotenoids are some of the most widely used natural products in sunscreen formulations [14,15]. Phenolics are well-recognized antioxidant agents that can act as free radical scavengers, thereby protecting the skin against solar radiation-induced oxidative stress [16], exhibiting antioxidant, anti-inflammatory, antitumor, antiproliferative, and antiangiogenic effects. Specifically, their antioxidant and anti-inflammatory properties have demonstrated efficacy in managing various dermatological conditions such as vitiligo or different types of lentigo [17,18]. In addition, phenolic compounds have been shown to significantly reduce UVB-induced oxidative damage by modulating the nuclear factor-erythroid-2-related factor 2/heme oxygenase-1 (Nrf2/HO-1) via the primary cellular antioxidant defense mechanism [19]. In this regard, the phenolic compound rosmarinic acid (RA) has gained attention as a potential photoprotective agent. Recent clinical studies have demonstrated that the association of 0.1% RA with ethylhexyl methoxycinnamate and avobenzone significantly enhances photoprotective efficacy, achieving an increase in sun protection factor (SPF) in human volunteers [20]. Flavonoids are the predominant group of phenolics that act by protecting plants from harmful UVB radiation. The presence of phenolic hydroxyl motifs confers a potent scavenger capacity to flavonoids, making them highly attractive for photoprotective applications. Recently, our group reported that morin (**1**) and its semi-synthetic derivatives, morin oxime and morin semicarbazone (**2**), obtained by classic Schiff-base synthesis, exhibited a promising potential to enhance photoprotection and mitigate UVB-induced skin damage through their antioxidant and anti-inflammatory activities. Specifically, pre-treatment with these compounds showed a photoprotective effect in UVB-exposed HaCaT keratinocytes, effectively reducing ROS, malondialdehyde (MDA), and interleukine-6 (IL-6) levels [21]. In addition to phenolics, carotenoids are a class of compounds widely used in photoprotection, capable of absorbing ultraviolet–visible (UV–Vis) radiation due to their highly conjugated double bonds [22,23]. Specifically, fucoxanthin (FX), a marine xanthophyll found in brown algae, has been reported as an antioxidant, anti-inflammatory, and anticancer agent. Regarding photoprotection, a previous study by our group showed the photoprotective effect of FX on UVB-exposed HaCaT cells by reducing ROS and IL-6 cytokine levels. Furthermore, the combination of FX with RA downregulated the NLRP3 inflammasome and upregulated the Nrf2 signaling pathway in UVB-irradiated HaCaT cells [24,25]. Notably, when these compounds were administered together, a synergistic antioxidant and anti-inflammatory effect was observed through activation of the ARE/Nrf2 transcription system [26].

Based on the urgent need to develop ecofriendly sunscreen ingredients that act as filters aids and stabilizing agents, and considering the therapeutical potential of both phenolics and carotenoids, we investigated the combined photoprotective effect of morin (**1**) or its derivative morin semicarbazone (**2**) with the carotenoid FX in HaCaT human keratinocytes exposed to UVB. We focused on their capacity to counteract the oxidative stress induced by UVB irradiation and inflammaging in HaCaT keratinocytes, exploring the underlying molecular mechanisms through the Nrf2/HO-1 antioxidant defense system.

## 2. Materials and Methods

### 2.1. Compounds and Experimental Design

The flavonoid morin (**1**) (M4008) and the carotenoid FX (F6932) were acquired from Sigma Aldrich (Saint Louis, MO, USA). Morin semicarbazone (**2**) was synthesized as described in our previous paper [16]. Based on the use of multi-target agents and the interest in the combination of natural products such as phenolics and carotenoids [24] with therapeutic properties, we proposed the combination of the phenolic compounds **1** or **2** with the carotenoid FX (designated as FX-**1** and FX-**2**, respectively). These combinations were evaluated at a 1:1 molar ratio using concentrations of 0.31, 0.625, 1.25, 2.5, and 5 µM.

### 2.2. Cell Culture

HaCaT keratinocytes were purchased from Cell Lines Service (CLS) GmbH (Cytion, Eppelheim, Germany). The medium used to maintain the cells was Dulbecco’s modified Eagle high glucose, with 2 mM L-glutamine (DMEM, GIBCO, Grand Island, NY, USA), and supplemented with 10% inactivated fetal bovine serum and a penicillin/streptomycin antibiotic cocktail (100 U/mL and 100 mg/mL, respectively) (BIOWEST, Nuaillé, France). Cells were maintained under standard incubation conditions (humidified atmosphere, 5% CO_2_, 37 °C).

### 2.3. UVB Irradiation Procedure

Human immortalized HaCaT keratinocytes were cultured until reaching 75–80% confluence and then pre-treated with the experimental compounds for 4 h. The medium was removed and a warm thin layer of phosphate-buffered saline (PBS, pH 7.2) was applied to prevent the cells from drying out and ensure uniform exposure. The light source provided a peak intensity at 302 nm, commonly used to simulate UVB radiation. A single UVB dose of 50 mJ/cm^2^ for 30 s was used to irradiate the cells by using a Bio-link^®^ crosslinker VILBER (Collégien, France). Finally, the PBS was removed and fresh complete medium added, and cells were incubated for the appropriate duration required for each specific assay [21].

### 2.4. ABTS Radical Scavenging Assay

The total antioxidant capacity of the FX and the combinations FX-**1** and FX-**2** was determined by using the free radical 2,2′-azino-bis (3-ethylbenzothiazoline-6-sulfonic acid) (ABTS•+) colorimetric method, as previously described [26]. Briefly, an ABTS•+ radical solution was added to the compounds and combinations prepared in 96-well plates at different concentrations (0–200 µM). For the combinations, a 1:1 molar ratio was maintained. Then, we monitored the absorbance at 734 nm by using a spectrophotometer (iMarkTM microplate reader, BIO-RAD, Hercules, CA, USA) for 6–7 min until the stabilization of absorbance. Trolox, a water-soluble analogue of vitamin E (α-tocopherol), was used as a control. The effective concentration 50% (EC50) was determined, and the results were expressed as TEAC (Trolox equivalent antioxidant capacity = EC50 sample/EC50 Trolox).

### 2.5. Determination of Cell Viability

The viability of keratinocytes upon exposure to FX, FX-**1**, and FX-**2** was determined by the resazurin assay. This method serves as an indirect measure of the toxicity of the tested compounds and UVB exposure by assessing the metabolic capacity of the cells to reduce resazurin to resorufin [27]. To evaluate the cytotoxicity of the compounds, cells were seeded into 96-well plates (100 µL/well) at 1.5 × 10^5^ cells/mL and incubated overnight at 37 °C. Afterwards, the cells were pre-treated with FX (0.31, 0.625, 1.25, 2.5, and 5 µM) and the combinations FX-**1** and FX-**2** at the same concentration range in a 1:1 ratio, for 24 h. On the other hand, to assess the photoprotective effect of the treatments against UVB radiation, the keratinocytes were pre-treated with FX (0.31, 0.625, 1.25, 2.5, and 5 µM) and the combinations FX-**1** and FX-**2** at the same concentration range in a 1:1 ratio, for 4 h. The cells were then washed twice (PBS, 4 °C), irradiated with UVB (50 mJ/cm^2^) for 30 s, and incubated with fresh medium for 24 h. For both experiments, the medium was removed and a resazurin solution (200 µL at 20 mg/mL) was added for 3 h. Finally, the absorbance was determined by using a spectrophotometer at 540 and 620 nm (iMarkTM microplate reader, BIO-RAD, Hercules, CA, USA).

### 2.6. Detection of Intracellular ROS Production

It is widely reported that intracellular ROS levels are increased upon UVB exposure. The most common method used to determine intracellular ROS production is the 2′,7′-dichlorodihydrofluorescein diacetate (DCF-DA) assay kit. Briefly, HaCaT cells were seeded in 96-well black plates (100 µL/well) at 2 × 10^5^ cells/mL and incubated for 24 h. Afterwards, the cells were pre-treated with FX (1.25, 2.5, and 5 µM) and the combinations FX-**1** and FX-**2** at the same concentration range in a 1:1 ratio for 4 h. Subsequently, the cells were washed twice (PBS, 4 °C), and PBS (50 µL/well) was added to carry out UVB irradiation (50 mJ/cm^2^) for 30 s. Then, the cells were incubated with a DCFH-DA solution (20 µM) for 45 min, according to the manufacturer’s instructions. Subsequently, the cells were washed twice (PBS, 100 µL/well). Fluorescence was monitored using a fluorescence reader (Sinergy H1, Biotek^®^, Bad Friedrichshall, Germany) at 485 nm (excitation) and 535 nm (emission).

### 2.7. Lipid Peroxidation

Exposure to UVB radiation may cause cell damage such as lipid peroxidation, which entails their transformation into malondialdehyde (MDA). The thiobarbituric acid reactive substance (TBARS) method was carried out to examine lipid peroxidation in HaCaT keratinocytes. Shortly, the cells were seeded in 6-well plates (2 mL/well) at 2.5 × 10^5^ cells/mL and incubated for 24 h. After a pretreatment with FX (5 µM), FX-**1** (5–5 µM), and FX-**2** (5–5 µM) for 4 h, the cells were washed twice with PBS and UVB irradiation (50 mJ/cm^2^) was performed for 30 s. Then, the cells were incubated in fresh medium overnight. Subsequently, the cells were collected by trypsinization to ensure the cell integrity and then centrifuged (1000 rpm, 4 °C, 5 min). The pellet was resuspended in a 0.5% thiobarbituric acid (TBA) solution dissolved in 20% trichloroacetic acid (100 µL) and incubated at 95 °C for 30 min in a thermostatic water bath. Next, centrifugation (10,000 rpm, 4 °C, 15 min) was carried out to discard cell debris. The absorbance of the supernatants was measured by using an iMark TM microplate reader (BIO-RAD, Hercules, CA, USA) at 540 nm. MDA levels were referred to μg of total protein.

### 2.8. Assessment of IL-6 Production

UVB radiation increases the generation of skin pro-inflammatory cytokines such as IL-6. In short, HaCaT human keratinocytes were seeded in 96-well plates (100 µL/well) at 1.5 × 10^5^ cells/mL, incubated for 24 h and treated with FX (1.25, 2.5, and 5 µM) and the combinations FX-**1** and FX-**2** at the same concentration range in a 1:1 ratio, for 4 h. Afterwards, the medium was removed and UVB irradiation (50 mJ/cm^2^) was performed for 30 s. Subsequently, the PBS was removed, and the cells were incubated with fresh complete medium for 24 h. Then, the supernatants were collected and kept at −80 °C until the IL-6 determination, which was carried out by using a commercial ELISA kit (Diaclone GEN-PROBE, Besançon, France). A microplate reader (iMarkTM microplate reader, BIO-RAD, Hercules, CA, USA) was used to measure the absorbance at 450 nm. The data were expressed as IL-6 synthesis normalized to cell viability to account for the cytotoxic effects of UVB irradiation.

### 2.9. Determination of MMP-9 Production

Matrix metalloproteinase-9 (MMP-9) is an enzyme widely known by degrading collagen and other extracellular matrix components. Its levels are overexpressed due to the increase in ROS levels induced by UV radiation. To analyze MMP-9, HaCaT keratinocytes were seeded into 6-well plates (2 mL/well) at 2.5 × 10^5^ cells/mL. After 24 h, the cells were incubated with compounds **1**, **2**, and FX (5 µM) and the combinations FX-**1** and FX-**2** at the same concentration in a 1:1 ratio for 4 h. Subsequently, the medium was removed, and UVB irradiation (50 mJ/cm^2^) was performed for 30 s. Following irradiation, the PBS was replaced by fresh medium (2 mL/well). After 24 h, the supernatants were kept at −80 °C. MMP-9 detection was carried out by ELISA (DY911-05). To measure the absorbance (450 nm), a spectrophotometer was used (iMarkTM microplate reader, BIO-RAD, Hercules, CA, USA). The data were expressed as MMP-9 production normalized to cell viability to account for the cytotoxic effects of UVB irradiation.

### 2.10. Protein Isolation and Western Blot Analysis

Keratinocytes were seeded in 6-well plates (2 mL/well) at 2.5 × 10^5^ cells/mL and pretreated with the compounds FX, **1**, and **2** (5 µM) and the combinations FX-**1** (5–5 µM) and FX-**2** (5–5 µM) for 4 h. Subsequently, the medium was removed, and a warm thin layer of PBS (50 µL/well) was added to carry out UVB irradiation (50 mJ/cm^2^) for 30 s. After that, PBS was removed and fresh complete medium was added for 24 h. Cells were collected along with the culture medium by trypsinization and centrifuged (1200 rpm, 4 °C, 5 min). The pellets were collected and mixed with 50 µL/sample of cold lysis buffer containing a cocktail of protease inhibitors (50 mM Tris-HCl pH 7.5, 8 mM MgCl_2_, 5 mM ethylene glycol bis(2-aminoethyl ether)-N,N,N′N′-tetraacetic acid, 0.5 mM EDTA, 1 mM phenylmethylsulfonyl fluoride, 0.01 mg/mL leupeptin, 0.01 mg/mL pepstatin, 0.01 mg/mL aprotinin, and 250 mM NaCl). The samples were sonicated on ice (95 °C, 15 min) and kept at −80 °C. The Bradford method was used to calculate 15 µg of total protein content for each cellular homogenate, which were separated into 10% acrylamide gel by using SDS-polyacrylamide-gel electrophoresis. Upon the proteins being transferred onto a nitrocellulose membrane, a blocking buffer (5% BSA) was incubated for 2 h to prevent non-specific binding, and membranes were then incubated overnight with specific primary antibodies at 4 °C: rabbit anti-Nrf2 (1:1000) (Santa Cruz Biotechnology, St. Louis, MO, USA), rabbit anti-HO-1 (1:1000) (Henzo^®^, Madrid, Spain), and rabbit anti-COX-2 (1:1000) (Cell Signaling, Danvers, MA, USA). Subsequently, the membranes were washed and incubated with horseradish peroxidase-linked anti-rabbit antibody or anti-mouse HRP secondary antibody (Cell Signaling, Danvers, MA, USA). Equal protein loading was measured using β-actin as a housekeeping gene (Sigma-Aldrich, St. Louis, MO, USA). An Amersham Imager 600 (GE Healthcare Life Sciences, Barcelona, Spain) was used for immunodetection, and Image Processing and Analysis (1.54r ImageJ^®^, Softonic, Bethesda, ML, USA) was used to analyze the data.

### 2.11. Flow Cytometry

The harmful effects of UV light on the skin, specifically its ability to induce cell death, are well-established. To investigate the protective effects of the compounds on UVB-induced cell death in HaCaT human keratinocytes, an Annexin V-FITC Apoptosis Detection Kit from BD Pharmingen (Franklin Lakes, NJ, USA) was used. Briefly, the keratinocytes were seeded in 6-well plates (2 mL/well) at 2.5 × 10^5^ cells/mL and incubated overnight. A pre-treatment with FX, **1**, and **2** (5 µM) and the combinations FX-**1** (5–5 µM) and FX-**2** (5–5 µM) were carried out for 4 h. Subsequently, the medium was removed and UVB irradiation (50 mJ/cm^2^) was performed for 30 s. Following irradiation, the cells were incubated with fresh medium for an additional 24 h. Afterwards, the cells were washed twice and collected along the culture medium by trypsinization, and the cell suspension was centrifuged (800 rpm, 4 °C, 4 min). The resulting cell pellet was incubated with annexin V-FITC and propidium iodide (PI) in darkness. A Beckman Coulter FC 500 MPL flow cytometer (Beckman Coulter, Hialeah, FL, USA) was used to measure fluorescence using MXP software examining 104 events, and data were analyzed using CXP software. The logarithmic scale FITC fluorescence cytogram allowed us to distinguish between FITC fluorescence versus PI fluorescence in the logarithmic scale, showing differences between viable, apoptotic, and necrotic cells.

### 2.12. Statistical Assessment

All results are expressed as the mean ± SEM. GraphPad Prism Version 5.00 software (GraphPad Software, Inc., San Diego, CA, USA) was used to carry out the statistical study. The Shapiro–Wilk test was used to determine the normal distribution of data. Statistical tests used for individual analysis are provided in the figure legends.

## 3. Results

### 3.1. Scavenging Activity

**Figure 1:**
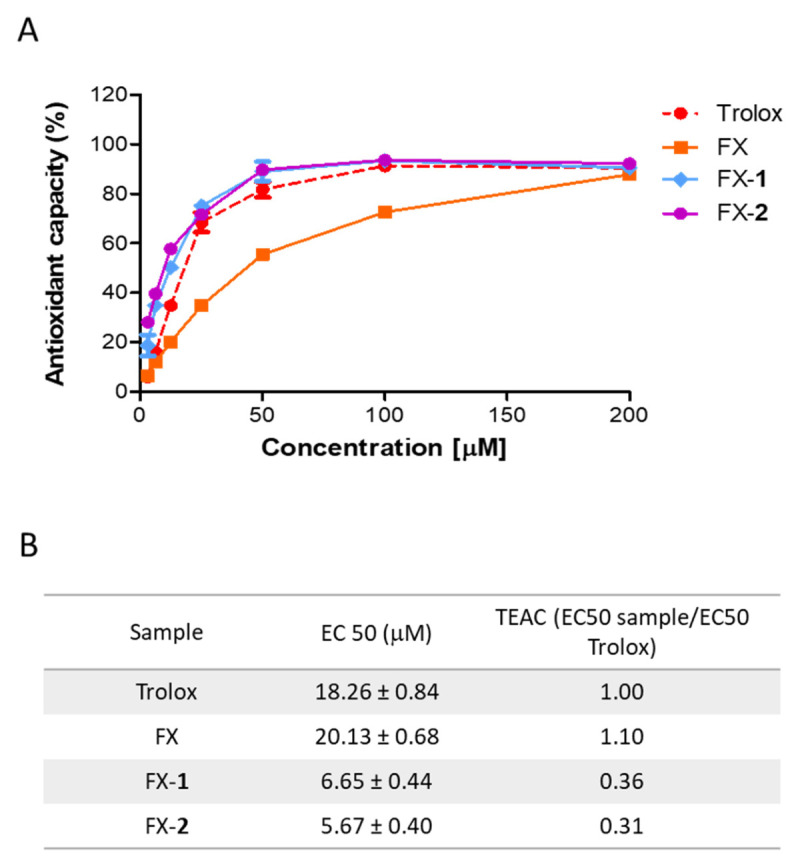
Antioxidant capacity of FX and the combinations FX-**1** and FX-**2**. (**A**) Scavenger effect tested at different concentrations (0–200 µM) by the ABTS method as described in the Materials and Methods section. (**B**) The EC50 was calculated, and data are expressed as TEAC. Results are the average of three independent experiments (n = 3).

The antioxidant capacity of the combinations FX-**1** and FX-**2** was analyzed by using the ABTS colorimetric assay. The ABTS•+ radical was exposed to a range of concentrations (0–200 µM) to determine the capacity of the treatments to donate electrons to this radical (Figure 1A). Commonly, this method is reported as TEAC, calculated as EC50 sample/EC50 Trolox (Figure 1B). In our previous study, morin (**1**) exhibited an EC50 of 10.75 ± 2.21 μM, and its derivative morin semicarbazone (**2**) showed an EC50 of 12.26 ± 0.92 μM, corresponding to TEAC values of 0.52 and 0.59 compared to Trolox, respectively [16]. In the present study, the reference control Trolox displayed an EC50 of 18.26 ± 0.84 μM, which is in accordance with our previous findings. FX showed an EC50 of 20.13 ± 0.68 μM, exhibiting an antioxidant capacity comparable to that of Trolox. Interestingly, the combination FX-**1** showed an EC50 of 6.65 ± 0.44 µM (TEAC = 0.36) and FX-**2** an EC50 of 5.67 ± 0.40 µM (TEAC = 0.31). These results indicate that approximately one-third of FX-**1** or FX-**2** is required to obtain a scavenging effect comparable to that of Trolox. Overall, these findings support that the scavenging activity of phenolics **1** and **2** is enhanced in the presence of FX compared to the compounds separately.

### 3.2. Cell Viability and Photoprotective Effect of the Compounds

**Figure 2:**
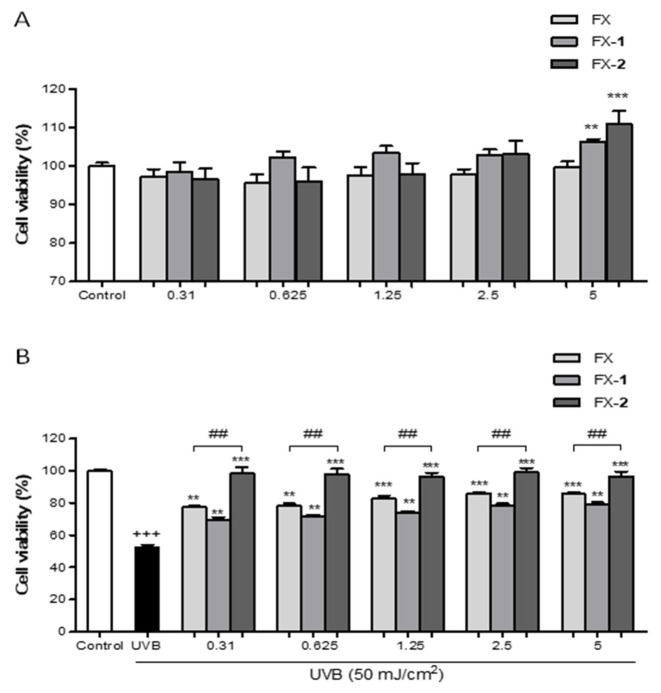
Cell viability of HaCaT human keratinocytes treated with FX, FX-**1**, and FX-**2** in the absence and presence of UVB light. (**A**) Cytotoxic activity of FX and the combinations FX-**1** and FX-**2** in non-UVB-exposed HaCaT cells. (**B**) Cytotoxic activity of FX and the combinations FX-**1** and FX-**2** in UVB-exposed HaCaT cells. Data are shown as the percentage of cell viability vs. untreated cells (n = 6). The mean was statistically different vs. the control (+++ *p* < 0.001; Student *t* test). The mean was statistically different vs. UVB-irradiated cells (** *p* < 0.01, *** *p* < 0.001; one-way ANOVA followed by Bonferroni’s). The mean was statistically different vs. FX treatment (## *p* < 0.01; Mann–Whitney *U*-test).

Firstly, the potential cytotoxicity of the phenolics was examined by using the resazurin assay (Figure 2). As previously reported, morin (**1**) and morin semicarbazone (**2**) exhibited no cytotoxicity effects on keratinocytes at concentrations up to 10 µM [16]. In the present study, FX and the combinations FX-**1** and FX-**2** also showed no cytotoxicity in the range of tested concentrations (0.31–5 μM). Interestingly, at the highest concentration tested (5 µM), FX-**1** and FX-**2** significantly increased the cell viability by 6.3% (*p* < 0.01) and 10.9% (*p* < 0.001), respectively, compared to the control group (Figure 2A).

In a separate set of experiments, the photoprotective role of compounds was evaluated in HaCaT human keratinocytes. HaCaT cells exposed to UVB radiation exhibited a 50% reduction in viability in comparison with non-irradiated control cells. Previously, we reported that morin (**1**) and morin semicarbazone (**2**) significantly increased cell viability in the concentration range of 5–10 µM. Similarly, in the present study, FX and the combinations FX-**1** and FX-**2** significantly increased the cell viability at all tested concentrations (0.31–5 µM). Specifically, FX and FX-**1** maintained over 70% viability starting from 0.31 µM (representing a reduction in cell death of more than 20% compared to the UVB control). Remarkably, FX-**2** maintained viability levels above 96%, exhibiting levels comparable to the non-irradiated control group. In addition, FX-**2** showed a significantly higher cell viability than FX alone at all tested concentrations (*p* < 0.01, respectively) (Figure 2B).

### 3.3. Antioxidant Effects of Treatments on ROS Production and Lipid Peroxidation

**Figure 3:**
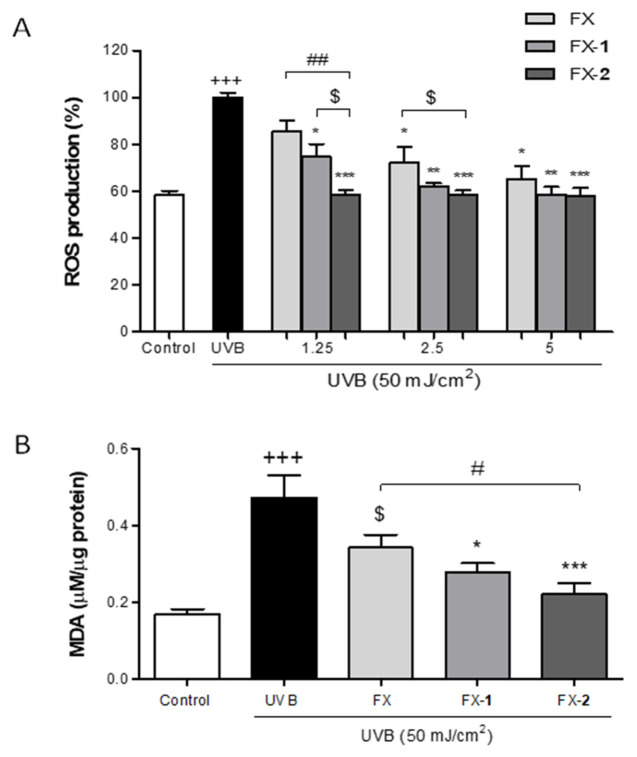
Effects of FX, FX-**1**, and FX-**2** against UVB-induced oxidative stress in HaCaT keratinocytes. (**A**) Intracellular ROS synthesis was assessed by using DCF-DA staining as described in the Materials and Methods section. (**B**) Lipid peroxidation was analyzed by measuring MDA production in UVB-exposed HaCaT cells. Results are presented as MDA mM/µg total protein. The bars correspond to the mean ± SEM (n = 4). The mean was statistically different vs. the control (+++ *p* < 0.001; Mann–Whitney *U*-test). The mean was statistically different vs. UVB-irradiated cells (* *p* < 0.05, ** *p* < 0,01, *** *p* < 0,01; Mann–Whitney *U*-test). The mean was statistically different vs. FX (# *p* < 0.05, ## *p* < 0.01; Mann–Whitney *U*-test). $ indicates tendency (*p* < 0.058).

Intracellular ROS levels in UVB-stimulated HaCaT human keratinocytes were determined by using DCFH-DA fluorescent staining. A bioactive and non-cytotoxic range of concentrations (1.25, 2.5, and 5 µM) was selected to carry out this study. Previously, we reported that morin (**1**) and morin semicarbazone (**2**) did not show a significant effect on UVB-induced ROS production [16]. In the present study, as expected, UVB exposure induced a statistical increase in ROS intracellular levels when compared to the non-irradiated control group (*p* < 0.001), which maintained basal ROS levels at 60% (Figure 3A). FX revealed a gradual decrease in ROS production, reaching statistical significance at the highest concentrations tested, 2.5 and 5 µM (*p* < 0.05 and *p* < 0.05, respectively), and restoring basal levels at 5 µM. FX-**1** pre-treatment reduced UVB-induced ROS production at three tested concentrations (*p* < 0.05, *p* < 0.01, *p* < 0.01, respectively), achieving ROS levels comparable to the control group from 2.5 µM. Interestingly, FX-**2** effectively reduced ROS production across the entire range (*p* < 0.001 for all concentrations), maintaining non-irradiated control ROS levels from the lowest tested concentration (1.25 µM). Furthermore, FX-**2** showed a statistically greater decrease in ROS levels in comparison with FX at 1.25 µM (*p* < 0.01) and a decreasing trend at 2.5 µM. Additionally, FX-**2** caused a reducing trend in ROS production compared to FX-**1** at 1.25 µM.

Upon UV exposure, an increase in intracellular ROS production may lead to lipid peroxidation, resulting in the formation of MDA. Thus, we further assessed the MDA levels in UVB-exposed HaCaT human keratinocytes as a marker of oxidative damage. Based on our previous findings, where morin (**1**) and morin semicarbazone (**2**) reduced the MDA levels by 30% and 50% at 5 µM, respectively, we evaluated FX and the combinations FX-**1** and FX-**2** at the same concentration. In the present study, UVB-irradiated HaCaT cells significantly showed higher levels of MDA compared to non-irradiated cells (*p* < 0.001) (Figure 3B). HaCaT keratinocytes pre-treated with FX (5 μM) notably reduced the MDA levels by 30%. The combination FX-**1** was able to significantly prevent MDA production by 40% (*p* < 0.05), while FX-**2** inhibited the MDA levels by 55% (*p* < 0.001). In addition, FX-**2** showed a significant reduction in MDA levels compared to FX alone (*p* < 0.05). These findings indicate that both combinations exert a more potent inhibitory effect on lipid peroxidation than the individual compounds, with FX-**2** showing the highest efficacy.

### 3.4. Assessment of the IL-6 and MMP-9 Inflammaging Mediators

**Figure 4:**
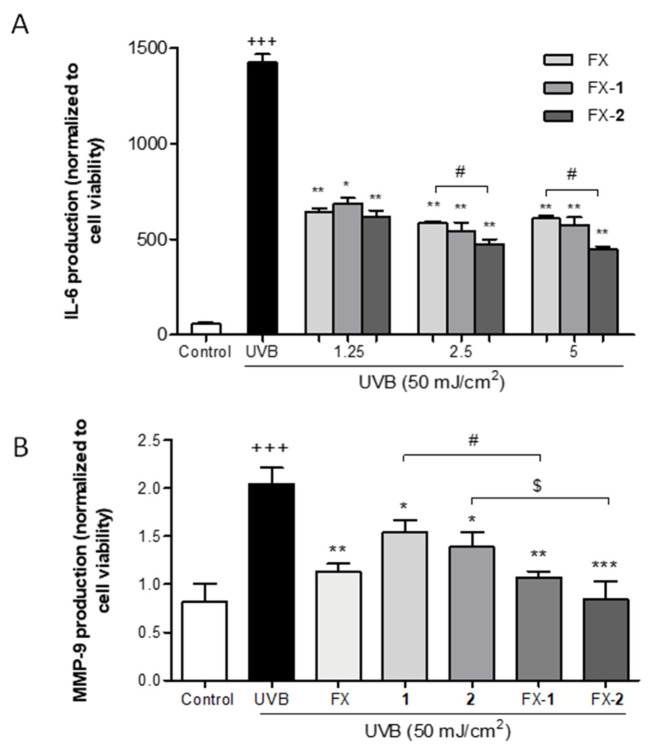
Effect of the treatments on IL-6 and MMP-9 inflammaging mediators in UVB-exposed HaCaT keratinocytes. (**A**) IL-6 production. (**B**) MMP-9 production. Results are expressed as IL-6 pg/mL or MMP-9 pg/mL normalized to cell viability, respectively. Both mediators were analyzed by ELISA as described in the Materials and Methods section. The vertical bars correspond to the mean ± SEM (n = 4). The mean was statistically different vs. the control (+++ *p* < 0.001; Mann–Whitney *U*-test). The mean value was statistically different vs. UVB (* *p* < 0.05, ** *p* < 0.01, *** *p* < 0.001), Mann–Whitney *U*-test). The mean was statistically different compared between groups (# *p* < 0.05; Mann–Whitney *U*-test). $ indicates tendency (*p* < 0.069).

The skin inflammatory process caused by UVB-induced ROS increase establishes a direct link between IL-6 and MMP-9 mediators. The anti-inflammatory activity of the carotenoid FX and the combinations FX-**1** and FX-**2** were examined by measuring IL-6 synthesis in UVB-irradiated HaCaT cells. Previously, we reported the anti-inflammatory activity of morin (**1**) and morin semicarbazone (**2**); specifically, 1 (2.5 and 5 μM) reduced IL-6 production by approximately 13% and 15%, respectively, while 2 (2.5 and 5 μM) achieved a 45% reduction at both tested concentrations (*p* < 0.05) [21]. According to our previous report, the exposure to UVB induced a significant 20-fold increase in IL-6 synthesis versus the non-irradiated control (*p* < 0.001) (Figure 4A). The pre-treatment with FX (1.25, 2.5, and 5 μM) decreased IL-6 production by 55% (*p* < 0.01), 59% (*p* < 0.01), and 57% (*p* < 0.01). The combination FX-**1** also reduced IL-6 production by approximately 60% at all three tested concentrations (*p* < 0.05, *p* < 0.01, and *p* < 0.01). Interestingly, FX-**2** (1.25, 2.5, and 5 μM) gradually decreased the production of this pro-inflammatory cytokine by 57% (*p* < 0.01), 67% (*p* < 0.01), and 70% (*p* < 0.01). Furthermore, FX-**2** significantly reduced IL-6 synthesis compared to FX alone at 2.5 and 5 μM (*p* < 0.05), emerging as the most potent combination.

Evidence indicates that elevated levels of IL-6 synthesis in the skin, often triggered by ROS, can induce the expression of MMP-9, leading to the degradation of multiple extracellular matrix components, including collagen IV and elastin, causing photoaging. Therefore, the MMP-9 levels were measured in UVB-exposed HaCaT human keratinocytes by the ELISA kit. The cells were pre-treated with FX, morin (**1**) or morin semicarbazone (**2**) (5 µM), or with the combinations FX-**1** and FX-**2** at the same concentration in a 1:1 ratio for 4 h, followed by UVB exposure. As expected, UVB significantly upregulated the MMP-9 levels in comparison to the non-irradiated cells (*p* < 0.001). Cells pre-treated with FX, **1**, or **2** significantly decreased the MMP-9 levels by 44% (*p* < 0.01), 25% (*p* < 0.05), and 32% (*p* < 0.05), respectively. Notably, the combinations FX-**1** and FX-**2** significantly decreased the MMP-9 levels by 48% (*p* < 0.01) and 59% (*p* < 0.001), respectively. These results confirmed that the combined treatments, particularly FX-**2**, exhibited a superior capacity to downregulate MMP-9 compared to the compounds used independently.

### 3.5. Modulation of the Nrf2/HO-1 Signaling and COX-2 Expression

**Figure 5:**
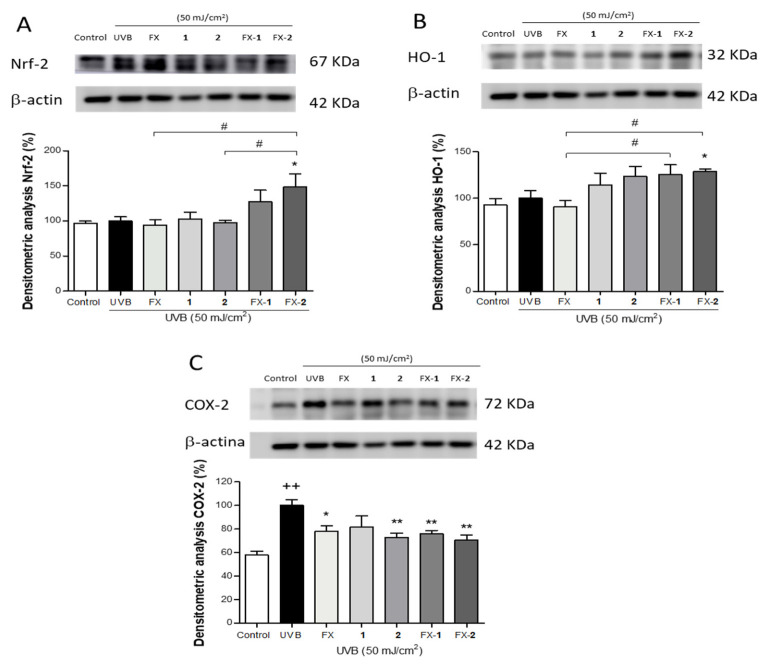
Effect of FX, **1**, **2**, and the combinations FX-**1** and FX-**2** on the protein levels. Protein expression was quantified in UVB-exposed HaCaT keratinocytes by Western blotting as described in the Materials and Methods section (**A**) Densitometric and representative Western blot analysis of Nrf2. (**B**) HO-1. (**C**) COX-2. The bars correspond to the mean ± SEM (n = 4). The mean was statistically different vs. the control (++ *p* < 0.01; Mann–Whitney *U*-test). The mean was statistically different vs. UVB-irradiated cells (* *p* < 0.05, ** *p* < 0.01). The mean was statistically different when compared between groups (# *p* < 0.05; Mann-–Whitney *U*-test).

To further elucidate the molecular mechanisms of FX, morin (**1**) and morin semicarbazone (**2**), and their combinations FX-**1** and FX-**2**, we investigated their impact on the Nrf2/HO-1 antioxidant via by using Western blotting. Keratinocytes were pre-treated with FX, **1**, and **2** (5 µM) and their combinations FX-**1** and FX-**2** at the same concentration in a 1:1 ratio for 4 h, and then exposed to UVB radiation. UVB irradiation did not significantly alter the basal Nrf2 expression levels (Figure 5A). The pre-treatment with FX, **1**, or **2** (5 µM) separately did not result in a significant change in the Nrf2 expression. In contrast, the cells treated with FX-**1** or FX-**2** (5/5 µM) were able to upregulate Nrf2 protein expression compared to the irradiated cells, with FX-**2** showing a statistically significant effect (*p* < 0.05). In addition, FX-**2** achieved a significantly higher upregulation of Nrf2 expression in comparison with FX and phenolic **2** (*p* < 0.05 and *p* < 0.05, respectively) when administered separately. Consistently, these effects on Nrf2 expression were accompanied by a notable increase in HO-1 expression in keratinocytes treated with the phenolics **1** and **2** separately, as well as with both combinations, with FX-**2** showing a statistically significant effect (*p* < 0.05). These data suggest a greater antioxidant effect of this combination (Figure 5B). To further address the anti-inflammatory role of FX, **1**, and **2** and their combinations (FX-**1** and FX-**2**), the expression of the pro-inflammatory enzyme COX-2, a key mediator in UVB-induced skin inflammation, was evaluated by Western blotting. Irradiated HaCaT cells exhibited a statistically increase in COX-2 expression compared to the control (*p* < 0.01) (Figure 5C). FX, **2**, FX-**1**, and FX-**2** significantly reduced the COX-2 levels (*p* < 0.05, *p* < 0.01, *p* < 0.01, and *p* < 0.01, respectively). Specifically, **2**, FX-**1**, and FX-**2** achieved a reduction of approximately 30%, effectively mitigating the pro-inflammatory response triggered by UVB.

### 3.6. Evaluation of UVB-Induced Cell Death

**Figure 6:**
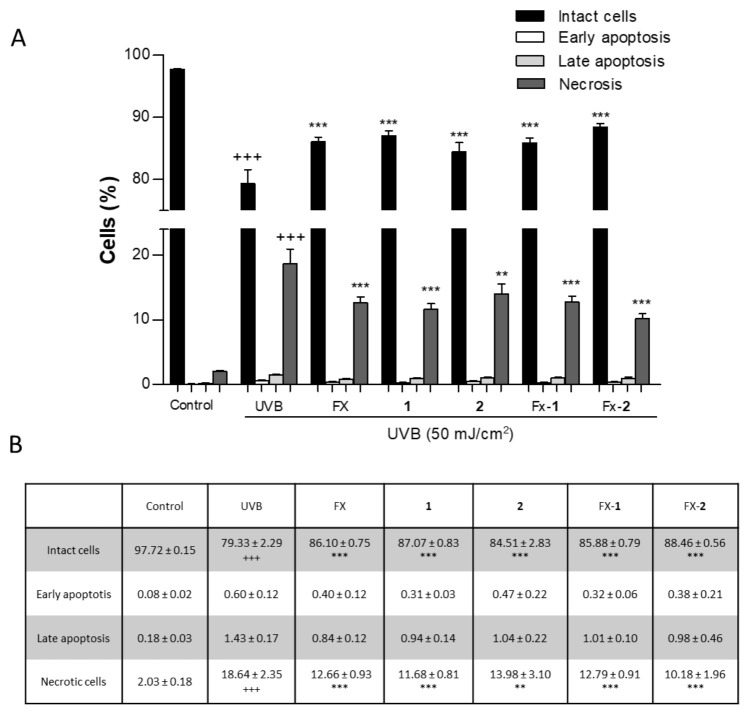
Effects of FX, **1**, **2**, FX-**1**, and FX-**2** on UVB-induced cell death in HaCaT keratinocytes. (**A**) Data of Annexin V-FITC assay are expressed as percentage of cells and bars correspond to the mean ± SEM (n = 6). (**B**) Quantification of the cell death expressed as the mean ± SEM according to the cell stage and treatment. Values were statistically different vs. the control in each respective cell phase (+++ *p* < 0.001; Student *t* test). Values were statistically different vs. UVB group in each respective cell stage (** *p* < 0.01, *** *p* < 0.001, Student *t* test).

UV radiation is widely known to induce cell death depending on the energy dose. In the present study, we examined the effect of UVB radiation on HaCaT viability and the protective effect of FX, morin (**1**), and morin semicarbazone (**2**), individually, using a non-cytotoxic concentration of 5 µM, as well as their combinations FX-**1** and FX-**2** at the same concentration in a 1:1 ratio (Figure 6A). As expected, the non-irradiated control cells showed a high viability of 97.72 ± 0.15%, whereas the exposure to UVB significantly reduced the cell viability to 79.33 ± 2.29% (*p* < 0.001), corresponding to an approximate 18% decrease. This reduction was accompanied by a significant increase in necrotic cells (18.64 ± 2.35%) (*p* < 0.001) compared to non-irradiated keratinocytes. Pre-treatment of HaCaT cells with FX, **1**, **2**, and FX-**1** for 4 h significantly increased the number of viable cells by 5–8% (25,000–40,000 cells) in comparison with the UVB-irradiated control. This effect was accompanied by a necrotic cell rate of up to 14%, representing a 4% reduction relative to the UVB-exposed cells. Interestingly, FX-**2** exhibited a cell viability of 88.46 ± 0.56% (representing an increase of more than 9% versus UVB group). This effect was accompanied by a necrotic rate of 10.18 ± 1.96% (corresponding to an approximate 9% reduction versus the UVB group) (Figure 6B). Therefore, FX-**2** was the most effective treatment, producing the most increased cell viability and the most reduced necrosis, protecting more than 50,000 cells from UVB-induced damage in this experimental model.

## 4. Discussion

UVB radiation is one of the most important factors in skin damage, contributing to photoaging, erythema, inflammation, or skin cancer. In response, photoprotection and cosmetic industries are focusing their efforts on introducing green and ecofriendly ingredients into sunscreens and other skin care products to mitigate environmental pollution, particularly as poorly biodegradable components accumulate in the marine environment [8]. Plants produce many secondary metabolites to protect themselves from UV radiation, which can be effectively used to protect human skin. In this regard, the use of natural antioxidant agents represents an effective strategy in the photoprotection field, because they protect cellular components from free radicals and thus reduce skin cell damage in an ecological and sustainable manner. Natural compounds, such as phenolics and carotenoids, have currently gained interest in the photoprotection field [28] due to their widely known antioxidant properties, radical scavenging activities, and DNA-protective effects. While these molecules are not intended to replace conventional UV filters, their structural features, such as chromophores and conjugated double bonds, may provide a supplementary UV-absorbing effect that complements traditional filters [14,21,29]. Therefore, understanding the molecular and cellular responses to UV radiation and how bioactive compounds act as adjuvants becomes crucial for developing effective photoprotective treatments. Furthermore, combining bioactive agents could offer enhanced efficacy through a multi-target approach compared to individual compounds. Thus, our study was conducted to validate the claim that the combination of the carotenoid FX with morin (**1**) (FX-**1**) or morin semicarbazone (**2**) (FX-**2**) have antioxidant, photoprotective, and anti-inflammatory properties in UVB-exposed HaCaT human keratinocytes. Our results showed that the percentage of viable HaCaT cells decreased upon exposure to UVB irradiation compared to non-irradiated cells. Consequently, a UVB dose of 50 mJ/cm^2^ was selected for the present study, as this dose maintains sufficient cell viability to allow for the observation of significant effects on other parameters and is consistent with doses used in previous studies [30,31].

In terms of oxidative stress, it is well-stablished that UVB exposure leads to an increase in intracellular ROS level in keratinocytes, promoting the degradation of proteins, DNA damage, cell death, and lipid peroxidation [32]. ROS-induced lipid peroxidation is one of the causes of early skin damage in photoaging, with MDA serving as a reliable biomarker for this process. In this study, the TBARS assay was employed as an indirect measure of lipid peroxidation by quantifying the MDA levels. While this colorimetric method is widely used due to its sensitivity, it is important to acknowledge that it can show cross-reactivity with other aldehyde species, which may limit its specificity [33]. Consistent with this approach, the relevance of MDA as a key marker has been further supported by recent ex vivo clinical studies using advanced methodologies. For instance, the high-performance liquid chromatography (HPLC)-TBARS protocol, which specifically isolates the MDA-TBA_2_ adduct, has been successfully employed to quantify lipid peroxidation in the stratum corneum of human participants, evaluating the efficacy of photoprotective formulations containing natural antioxidants like RA [34].

Scientific evidence has revealed that the use of antioxidant agents protects against UVB radiation-induced oxidative stress. In this regard, previous studies have reported that morin (**1**) protects skin cells, including keratinocytes and fibroblasts, against UV-induced damage, by inhibiting intracellular ROS production [35,36]. In addition, our group has previously demonstrated the antioxidant and photoprotective activity of morin (**1**) and its derivative, morin semicarbazone (**2**), acting as scavengers of free radicals and reducing ROS production and MDA levels, as a consequence of lipid peroxidation. Additionally, both compounds **1** and **2** exhibited UV–Vis absorption spectra (200–500 nm), which suggests their potential as organic filters [21]. On the other hand, FX can act as a scavenger of free radicals and downregulates oxidant parameters induced by UVB radiation such as ROS and MDA in vitro and in vivo experimental models [37,38,39,40]. Consistent with these studies, our data demonstrated that the combinations FX-**1** and FX-**2** protected HaCaT keratinocytes from UVB-induced oxidative stress and prevented lipid peroxidation, showing a greater effect than FX alone. This enhanced effect could be related to their potent scavenging activity, which was greater than that of the individual compounds, and was even three times higher than the reference control, Trolox.

Furthermore, our findings demonstrate that all treatments effectively increased cell viability following UVB exposure. Notably, FX-**2** exhibited an enhanced photoprotective effect compared to FX alone. While FX and FX-**1** partially rescued cells from UVB-induced damage (maintaining 70% viability), the FX-**2** combination almost entirely preserved cell viability, achieving levels comparable to the non-irradiated cells (96%) (Figure 2B). Our flow cytometry analysis further elucidated the mechanisms underlying the observed recovery in cell viability, confirming that UVB-induced damage primarily triggers necrotic pathways in HaCaT cells. It is worth highlighting that the more pronounced impact observed in the resazurin assay (approximately 50% reduction in metabolic activity) compared to the necrotic population detected via flow cytometry (approximately 20%) suggests that UVB radiation induces a profound metabolic decline well before the loss of membrane integrity [41,42]. In this context, while individual treatments and the FX-**1** combination provided a moderate protective effect, FX-**2** emerged as the most active photoprotective agent, significantly reducing the necrotic population (Figure 6).

In terms of photoaging, excessive UV exposure increases intracellular ROS, which triggers the MAPK signaling pathway, promoting low-grade inflammation or inflammaging [43,44,45]. This process leads to the activation of AP-1 and NF-kB transcription factors, upregulating the expression of pro-inflammatory mediators (IL-6, IL-1β, TNF-α) and MMPs (MMP-1, MMP-3, MMP-9) [46] while downregulating TGF-β expression, which is involved in collagen synthesis [47]. IL-6 and MMP-9 have been described as pivotal parameters of UVB-induced inflammation, redness, tissue breakdown, and wrinkle formation. Furthermore, a single exposure to UV irradiation may cause near-complete loss of pro-collagen synthesis in healthy adults, which persists for up to 24 h, followed by recovery 48–72 h post exposure [48,49]. When repeated over time, this effect is the main cause of photoaging. Therefore, inhibition of UVB-induced IL-6 and MMP-9 may play critical roles in preventing photoaging. In our previous study, morin (**1**) only slightly reduced the IL-6 levels whereas morin semicarbazone (**2**) decreased this parameter by 50% at 5 µM. In our present study, the combinations of these phenolics with the carotenoid FX (FX-**1** and FX-**2**) resulted in a much greater reduction in IL-6 levels, with FX-**2** being the most active combination, achieving a 70% decrease. On the other hand, we observed that UVB exposure increased MMP-9 expression in HaCaT cells. In this regard, our results revealed that FX, **1**, and **2** caused a marked reduction in MMP-9 levels. Interestingly, while FX-**1** showed similar effects than FX alone, FX-**2** significantly reduced the MMP-9 levels to levels comparable to the non-irradiated control cells. These findings support the hypothesis that a combined active treatment improves the bioactivity profile compared to the individual compounds [30]. Thus, although further studies are needed, the combined effects of FX-**2** reducing both IL-6 and MMP-9 support, at least in part, that FX-**2** may inhibit the activation of the ROS-induced MAPK pathway, thereby mitigating UVB-induced inflammatory responses and inflammaging. Exposure to UVB also induces the expression of genes linked to inflammation and oxidative stress [50,51,52]. Elevated levels of COX-2 have been observed in keratinocytes exposed to UVB [53,54]. In accordance with our IL-6 results, all treatments significantly reduced COX-2 expression in a similar way. On the other hand, Keap1/Nrf2/HO-1 via is the main mechanism involved in the regulation of UVB radiation-associated oxidative stress [55]. Upon oxidative stress, Nrf2 is phosphorylated by MAPKs, acting as a redox-sensitive transcription factor that undergoes nuclear translocation and promotes the transcriptional activation of its target genes such as HO-1. More specifically, phosphorylation of Nrf2 triggers a conformational change that leads to its dissociation from its natural inhibitor Keap1 dimer. Previous authors have found that the binding sites Arg415, Arg483, Tyr525, Tyr572, and Ser602 play a significant role in Keap1/Nrf2 complex stability [26,56], with the residues Arg415 and Tyr525 being key molecular targets for many molecule suppressors in the Keap1 binding pocket. Along this line, FX has been demonstrated to be the target of Keap1, blocking the formation of the Keap1/Nrf2 complex by hydrogen bonding at the residues Arg415 and Tyr525 of the Keap1 factor at concentrations ranging from 5 to 20 µM, thereby promoting Nrf2 activation in human keratinocytes and rat neurons [32,57]. Therefore, we herein selected a concentration of 5 µM to carry out our study, ensuring the use of the minimal active concentration of the treatments. In our study, FX at 5 µM did not increase Nrf2 or HO-1 expression, likely due to the low concentration used. Regarding morin (**1**), previous in vitro and in vivo experimental approaches have revealed that **1** is able to induce the expression of Nrf2 [58,59,60,61,62]. Nevertheless, this is the first study to investigate these effects in the context of photoprotection, using UVB-exposed HaCaT keratinocytes. Recently, **1** has been reported to show hydrogen bond interaction with Ser363 and hydrophobic interactions with Tyr334, Tyr572, Arg415, Gly462, Gly509, Ala556, and Ser602 of the protein Nrf2 [63]. This distinct binding profile compared to FX supports a complementary mechanism for upregulating the Nrf2 pathway. Interestingly, while **1** and **2** alone at the tested concentrations did not significantly increase the expression of Nrf2, they were able to induce HO-1 levels. Since HO-1 is a direct downstream target of Nrf2, its upregulation serves as a reliable indirect marker for Nrf2 transcriptional activity and nuclear translocation in this model. Furthermore, FX-**1** and FX-**2** were able to upregulate both Nrf2 and HO-1 expression at these low concentrations. The fact that these combinations increased the expression of these markers while the individual compounds had a more limited impact, highlights the enhanced protective response triggered by the combined treatment, even under restricted exposure times. These effects suggest that the combined use of bioactives with different molecular targets can achieve a potent protective effect at minimal doses, representing a useful strategy for skin protection. Taken together, our data support the efficacy of the multitarget combinations FX-**1** and FX-**2** for their use as potential green adjuvants in sunscreen formulations.

The present study has several strengths, primarily evaluating the potential of combinations of pure compounds (morin or morin semicarbazone with FX) as antioxidant and anti-inflammaging agents. Our findings provide preliminary evidence suggesting that these specific combinations could potentially act as protective adjuvants against UVB-induced damage in HaCaT keratinocytes. However, some limitations must be acknowledged. As these are preliminary in vitro results, further studies are required to evaluate essential parameters for topical formulations such as the photostability of the compounds and their skin irritation potential. Furthermore, future investigations should explore the combination of these natural molecules with conventional UV filters; this approach could potentially allow for a reduction in the concentration of conventional filters in the final formula and add multifunctional benefits. Finally, additional in vivo studies are necessary to confirm clinical relevance and determine the SPF of these combinations in human subjects.

## 5. Conclusions

In the present study, our findings suggest for the first time that the combinations FX-**1** and FX-**2** exhibit improved photoprotective effects compared to the individual compounds, with FX-**2** showing the most promising profile on UVB-exposed HaCaT keratinocytes. Specifically, the FX-**2** combination was able to effectively reduce UVB-induced cell death and restore ROS and MDA production to basal control levels. Furthermore, FX-**2** showed potential anti-inflammaging properties by reducing IL-6, MMP-9, and COX-2 mediators, and antioxidant activity through the upregulation of Nrf2/HO-1 signaling pathway. Thus, we propose FX-**1**, and particularly FX-**2**, as potential agents for further evaluation in photoprotective formulations. These combinations represent promising candidates to be used as adjuvants against UVB-induced skin damage such as photo-aging, inflammaging, or skin cancer. However, further research is required to ensure their photostability, safety, and in vivo efficacy.

## Data Availability

The original contributions presented in this study are included in the article. Further inquiries can be directed to the corresponding authors.

## Abbreviations

The following abbreviations are used in this manuscript:

ABTS	2,2′-Azino-bis (3-ethylbenzothiazoline-6-sulfonic acid
COX-2	Cyclooxygenase-2
DCF-DA	2′,7′-Dichlorodihydrofluorescein diacetate
EC50	Effective concentration 50%
FX	Fucoxanthin
HO-1	Heme oxygenase-1
IL-6	Interleukin-6
MDA	Malondialdehyde
MMP-9	Matrix metalloproteinase-9
Nrf2	Nuclear factor erythroid 2-related factor 2
PBS	Phosphate-buffered saline
PI	Propidium iodide
ROS	Reactive oxygen species
SEM	Standard error of the mean
TBA	Thiobarbituric acid
TBARS	Thiobarbituric acid reactive substance
TCA	Trichloroacetic acid
TEAC	Trolox equivalent antioxidant capacity
UV	Ultraviolet
Vis	Visible
